# Chemical Analysis of the Chinese Liquor Luzhoulaojiao by Comprehensive Two-Dimensional Gas Chromatography/Time-of-Flight Mass Spectrometry

**DOI:** 10.1038/srep09553

**Published:** 2015-04-10

**Authors:** Feng Yao, Bin Yi, Caihong Shen, Fei Tao, Yumin Liu, Zhixin Lin, Ping Xu

**Affiliations:** 1 State Key Laboratory of Microbial Metabolism, and School of Life Sciences & Biotechnology, Shanghai Jiao Tong University, Shanghai 200240, People's Republic of China; 2 National Engineering Research Center of Solid-State Brewing, Luzhou 646000, People's Republic of China; 3 The Instrumental Analysis Center, Shanghai Jiao Tong University, Shanghai 200240, People's Republic of China

## Abstract

Luzhoulaojiao liquor is a type of Chinese liquor that dates back hundreds of years, but whose precise chemical composition remains unknown. This paper describes the screening of the liquor and the identification of its compounds using comprehensive two-dimensional gas chromatography/time-of-flight mass spectrometry (GC × GC/TOF-MS). Samples were prepared by both liquid-liquid extraction and solid-phase microextraction, which facilitated the detection of thousands of compounds in the liquor, thus demonstrating the superior performance of the proposed method over those reported in previous studies. A total of 320 compounds were common to all 18 types of Luzhoulaojiao liquor studied here, and 13 abundant and potentially bioactive compounds were further quantified. The results indicated that the high-performance method presented here is well suited for the detection and identification of compounds in liquors. This study also contributes to enriching our knowledge of the contents of Chinese liquors.

Chinese liquors are among the oldest distillates in the world, dating back about 6,000 years^1^. The annual consumption of Chinese liquors in general is over four million kiloliters^2^. Like other distillates, Chinese liquors are usually fermented from grains for several months or years. The fresh fermentative liquors are then distilled and aged for a long time to enhance the bouquet. The particular brewing processes (fermentation, distillation, and aging) leads to the formation of a complex set of components^2^.

Luzhoulaojiao liquor is historically one of the most famous Chinese liquors whose fame has spread worldwide since its award of the gold medal at the Panama-Pacific International Exposition of 1915 in San Francisco^3^. Furthermore, Luzhoulaojiao liquor has enjoyed a lofty reputation in China since it was named among the four most famous Chinese liquors at the first Liquor Tasting Conference in 1952 in Beijing (the three others being Xifeng, Fen, and Moutai). Like other Chinese liquors, Luzhoulaojiao liquor consists of a complex mixture of compounds with a wide range of different physicochemical properties^1^, which may contribute to the flavor and bioactivity of the liquor. The identification of these components is therefore an important step in understanding and improving the distillates.

Currently, the chemical analysis of liquor components is performed by gas chromatography-olfactometry, or by gas chromatography coupled to mass spectrometry after sample extraction^4^. However, distinguishing the compounds in a complex matrix such as Chinese liquor proves difficult when using traditional one-dimensional gas chromatography, even with highly efficient columns and temperature gradients^5^. For the chemical analysis of complex mixtures, advanced chromatography—such as comprehensive two-dimensional gas chromatography (GC × GC) coupled with time of flight mass spectrometry (TOF-MS)—is the preferred approach^6^. Moreover, the effectiveness of GC × GC is broadly accepted for the detection of volatile compounds, which are the dominating trace composition in Chinese liquor^1^,^7^. The international standard databases (MAINLIB and REPLIB) facilitate detection and qualification, due to the use of electron impact ionization (EI), which produces repeatable and interpretable spectra^8^,^9^,^10^. However, reports on the application of GC × GC for the chemical analysis of Chinese liquors are few in the literature^5^,^11^,^12^, and this technique was first applied on a Chinese liquor (Moutai) in 2007 only^5^. However, the performance of the applied method (GC × GC/TOF-MS coupled to liquid-liquid extraction (LLE)) was limited by the capacity of LLE, whereby only 528 of the compounds in Moutai were extracted. Improving the chemical analysis needs to improve the associated sample extraction methods. Common among these are LLE, solid phase extraction, solid-phase microextraction (SPME), and stir bar sorptive extraction^13^. Liquid-liquid extraction using organic solvents has been shown to be a suitable method for the concentration of target compounds in Chinese liquor, readily extracting most of the volatile compounds^5^. Solid-phase microextraction is based on the adsorption of SPME fibres^14^ and is complementary to LLE. As well as being a solvent-free approach, it offers reproducibility, sensitivity, and selectivity^2^. The extraction biases of LLE and SPME are different, leading to the identification of different sets of compounds. In a previous study of distilled liquors indeed, LLE was found to extract a greater number of higher alcohols, while SPME was to be more sensitive to esters and acids^15^. Using both methods in combination may increase the number of compounds that can be identified and compared.

In this study, comprehensive two-dimensional gas chromatography/time-of-flight mass spectrometry (GC × GC/TOF-MS) with both LLE and SPME is shown to improve the chemical analysis of liquors. Applying this approach to Luzhoulaojiao liquors reveals certain abundant compounds, potentially bioactive compounds, and mark compounds, which may allow the brand of the Luzhoulaojiao liquor to be distinguished. Quantification of some interesting compounds was also performed to verify the proposed approach.

## Results

### Contour plots of the liquor extracted by LLE and SPME

**Figure 1:**
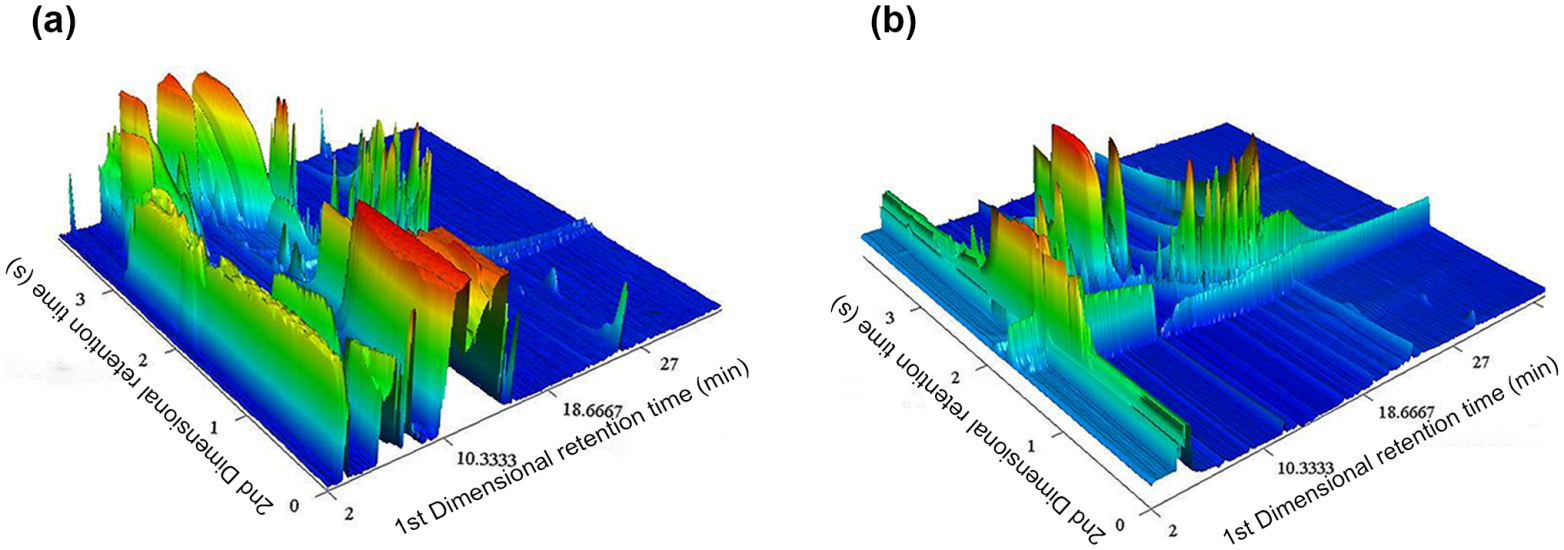
GC × GC/TOF-MS chromatography 3D plot of the extracts of Luzhoulaojiao liquor L9 extracted by liquid-liquid extraction (a) and solid-phase microextraction (b). The 1st dimension time is the retention time in the vapor pressure dimension and the 2nd dimension time is the retention time in the medium dimension, and the plots of abundant compound were red while the ones with low concentration were blue. A larger number of peaks were detected from the extracts by LLE than that by SPME. By performing SPME, some peaks invisible using LLE could be detected due to the specificity of the fiber. Usage of SPME and LLE can facilitate comprehensive chemical analysis in the Luzhoulaojiao liquor.

Eighteen types of Luzhoulaojiao liquor were extracted by LLE using anhydrous ether and *n*-pentane as solvents, as well as by SPME. Three-dimensional analysis plots of the Luzhoulaojiao liquor L9 are shown in Figure 1a and Figure 1b, as obtained by comprehensive two-dimensional GC × GC/TOF-MS. The three-dimensional chromatograms illustrate the complexity of the samples analyzed. The two extraction methods lead to different results, with 2,482 and 2,178 chromatography peaks detected in the Luzhoulaojiao L9 extracted by LLE and SPME, respectively.

### Analyses of various Chinese liquors

**Figure 2:**
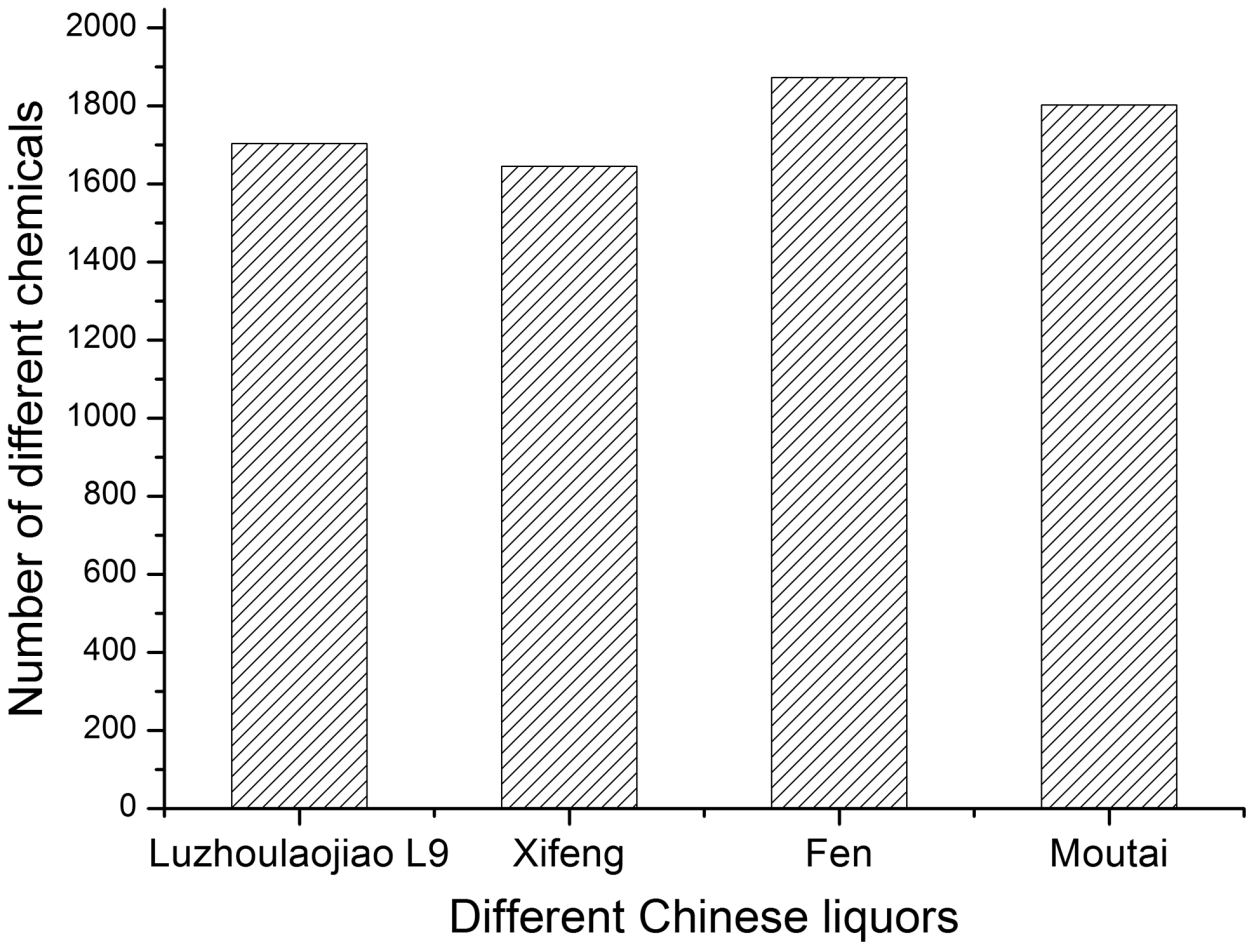
Components detected in different Chinese liquors. Chinese liquors Xifeng, Fen, and Moutai were analyzed by GC × GC/TOF-MS. GC × GC/TOF-MS was demonstrated as a good method for compound analysis of Chinese liquors because over 1,600 compounds could be detected by this method.

To test the method developed here, Luzhoulaojiao liquor L9 (a type of Luzhoulaojiao liquors easily purchased) and three other Chinese liquors (Xifeng, Fen, and Moutai) were analyzed by GC × GC/TOF-MS using LLE and SPME. Figure 2 compares the number of different chemicals found in each of these, with at least 1,600 compounds detected in each liquor and over 1,800 in Moutai (Supplementary Data S1). Considering that only 528 compounds were detected in the previous LLE–GC × GC/TOF-MS study of Moutai, these results highlight the synergistic improvements afforded by LLE and SPME.

### Compound diversity in Luzhoulaojiao liquors

To the best of our knowledge, Luzhoulaojiao liquors have not been submitted to date to a comprehensive chemical compound screening. This was performed here using TOF-MS spectral databases including MAINLIB and REPLIB. The signal to noise criterion was adjusted to “≥50” to include most of the peaks and the identification acceptance criterion was set as “similarity > 600”. This led to the identification of more than 1,300 compounds in each type of Luzhoulaojiao liquor. Luzhoulaojiao liquor L17 was found to be the richest by this measure, with about 2,400 compounds detected.

### Common compounds in 18 types of Luzhoulaojiao liquor

Identifying the compounds that are common to all the Luzhoulaojiao liquors is of interest as they form the chemical signature of the brand of Luzhoulaojiao liquor. Comparing the results obtained for 18 Luzhoulaojiao variants revealed 320 common compounds (Supplementary Data S2). In contrast, some compounds were only found in a few liquor samples—2-pentanamine for example, which was only found in liquor L1. Differences in the raw materials, storage, and brewing processes used (fermentation, distillation, and aging) as well as the blending of different Luzhoulaojiao liquors could contribute to some of these differences. From the extracts prepared by LLE and SPME, the most abundant class of compounds common to all the Luzhoulaojiao liquors were esters, while a large number of compound classes including alcohols, organic acids, etc. were also identified.

A total of twenty-seven of the compounds common to all the Luzhoulaojiao liquors tested are alcohols (Supplementary Table S1), most of which are saturated straight-chain alcohols ranging from 1-butanol to 1-nonanol. Unsaturated, aromatic, and saturated branched alcohols were also found, as were monohydric and polyhydric alcohols.

Nineteen organic acids (Supplementary Table S2) common to all 18 types of Luzhoulaojiao liquor were detected. Most of these are saturated straight-chain fatty acids ranging from acetic acid to undecanoic acid, while unsaturated, aromatic, and saturated branched organic acids were also among the common compounds.

The most versatile class of compounds identified was esters, about one hundred of these being common to all 18 types of Luzhoulaojiao liquor (Supplementary Table S3). Ethyl esters were the most numerous, including a series of saturated straight-chain fatty acid ethyl esters ranging from ethyl butyrate to ethyl decanoate. Unsaturated fatty acid ethyl esters and saturated branched acid ethyl esters were also detected—among which ethyl hexanoate was the most abundant—as well as a number of methyl, butyl, and hexyl esters.

The compounds common to all 18 types of Luzhoulaojiao liquor also include twenty ketones, thirteen aldehydes and ten acetals, both saturated and unsaturated (Supplementary Table S4). Most of the aldehydes are furfurals and methylals.

Finally, the list of common compounds also includes twenty-eight nitrogen- and seven sulfur-containing compounds (Supplementary Table S5). Pyridine and pyridine derivatives are the major nitrogen-containing compounds, while the seven sulfur-containing compounds include dimethyl disulfide and butanethioic acid S-methyl ester.

### Significant and distinct compounds in Luzhoulaojiao liquor

Luzhoulaojiao liquor is a typical representative of strong-aroma liquors, for which the most potent compounds are ethyl hexanoate, ethyl acetate, and ethyl lactate (Table 1). In Luzhoulaojiao liquor L9, the concentration of these abundant compounds was found to be 2,221 mg/L, 693 mg/L, and 316 mg/L, respectively (Table 2). Moreover, Table 3 lists the tens of potentially distinctive compounds found in Luzhoulaojiao liquors.

According to their relative contents (peak area), the most significant compound in Luzhoulaojiao liquor L9 and Xifeng (strong-aroma style) is ethyl hexanoate, whose concentration in the aforementioned liquors is much higher than it is in Fen (light-aroma style) and Moutai (soy sauce–aroma style). This is in agreement that ethyl hexanoate is the most abundant aroma compound in strong-aroma Chinese liquors^1^. Meanwhile, more ethyl acetate was detected in Fen and there was more furfural in Moutai.

The 320 compounds common to all 18 types of Luzhoulaojiao also include some potentially valuable aroma and bioactive substances. Some of these are listed in Tables 4 and 5. The aroma (such as ethyl hexanoate and ethyl lactate) and potentially bioactive compounds (such as 2,4-decadienal and acetophenone) detected in this study may serve as good targets for further investigations of the unique features of Luzhoulaojiao liquor.

## Discussion

Roughly ten years have elapsed since comprehensive GC × GC/TOF-MS was first applied for the chemical analysis of alcoholic drinks^16^, specifically for amino acid, and methoxypyrazines analysis in wines and beers^6^,^16^. In 2007, a similar approach was used to study samples of a Chinese liquor (Moutai) extracted by LLE leading to the identification of 528 compounds^5^, and demonstrating the power of this technique (viz. GC × GC/TOF-MS coupled to LLE) for chemical analysis. In this study, SPME and LLE were combined for enhanced extraction and a more complete characterization of the compounds in Chinese liquors.

Figure 1 compares 3D GC × GC plots obtained using different sample extraction methods. More peaks are observed in the LLE than in the SPME spectrum (Figure 1). The sample obtained by LLE using anhydrous ether and *n*-pentane as solvents contains most of the volatile compounds of the liquor, as subsequently detected by GC × GC. The selectivity SPME fiber (divinylbenzene/carboxen on polydimethylsiloxane (DVB/CAR on PDMS), 30/50 μm) is generally preferred to prepare liquor samples^2^,^17^. To perform SPME with high efficiency, the liquor samples were diluted to an appropriate concentration of 10–20% (v/v) ethanol, as recommended in the literature^2^,^13^. As expected, some specific compounds not seen in Figure 1a (LLE) are observed in Figure 1b (SPME). Further analysis reveals further differences in the number of compounds detected using LLE and SPME. Indeed, for Luzhoulaojiao liquor L9, both the LLE and SPME samples contain more than one hundred compounds such as ethyl hexanoate, ethyl acetate, and ethyl lactate. However, most of these are only detected in one or other of the samples. For instance, linoleic acid ethyl ester, hexadecanoic acid, and decanoic acid are not detected in the SPME sample. Meanwhile, no lactic acid, oleic acid, or octanol is identified in the Luzhoulaojiao liquor L9 extracted by LLE. These results highlight the selectivity of LLE and SPME towards specific compounds, suggesting that combining the two techniques affords a more complete identification of the unknown compounds found in Luzhoulaojiao liquors.

It is notable that over 1,300 compounds were detected for each type of the Luzhoulaojiao liquor investigated here, and that most of these are also found in other Chinese liquors. Moreover, many of these compounds are detected here in Chinese liquors for the first time. The volatiles in different Luzhoulaojiao liquors have been studied previously. For example, in 2014, 31 compounds were detected in 12 liquors from Luzhou Co., Ltd. by GC-MS^18^. Moreover, Guojiao1573, a type of Luzhoulaojiao liquor was extracted by SPME, within which 86 compounds were detected^19^. Many more compounds were detected in Luzhoulaojiao liquors in the present study, demonstrating the value of GC × GC/TOF-MS analysis coupled to LLE and SPME in this context. In a previous study reported in 2007, 528 compounds were detected in Moutai liquor. Here, more than 1,800 compounds were detected in the same liquor, demonstrating the gain in efficiency afforded by the method developed herein. It is reasonable to suggest that the approach put forward in this study will be integral to decomposing the complex compound matrix of Chinese liquor.

A total of 320 compounds were found to be common to all the Luzhoulaojiao liquors investigated here. Twenty-four of these are known to be bioactive^20^,^21^,^22^,^23^,^24^,^25^,^26^,^27^,^28^,^29^,^30^,^31^,^32^,^33^,^34^,^35^,^36^,^37^. Thirteen of the compounds in the extracts of Luzhoulaojiao liquor L9 were quantified, as listed in Table 2. The most abundant are ethyl hexanoate, ethyl lactate, and ethyl acetate, at 2,221 mg/L, 316 mg/L, and 693 mg/L, respectively. These concentrations are consistent with those measured previously^1^, highlighting the reliability of the developed method. After careful comparison with previously reported data, several compounds were identified here including butyl caprylate, furfuryl hexanoate, and phenylethyl butyrate that possess similar chemical properties as flavor compounds identified elsewhere in Chinese liquors. These may therefore serve as distinct markers for Luzhoulaojiao liquor.

The differences in the nature and number of compounds detected in the different types of Luzhoulaojiao liquor are noteworthy. As is well known, Chinese liquors go through a complicated preparation process, which determines their composition. One may logically speculate that variations may arise from the microbial density of the fermentation pits, the raw materials, and the production process. Changes in fermentation conditions are indeed known to lead to changes in composition, phenolic compounds for example being the direct result of the degradation of raw materials. Furthermore, the aging, distillation, blending, and storage processes can also alter the composition of Luzhoulaojiao liquor.

In summary, an analytical method, namely GC × GC/TOF-MS coupled with LLE and SPME, has been developed for compound identification in Chinese liquors. Thereby, over 1,300 compounds were identified in each of the 18 Luzhoulaojiao liquors analyzed, of which 320 were common to all extracts, many with interesting properties. Indeed, twenty-four compounds were found, which had previously been reported to be bioactive, and hundreds of compounds were identified that might contribute to the special flavor of Luzhoulaojiao liquor. These compounds should form the object of further research. This study may help to enrich our knowledge of the components of Chinese liquors, while the analytical method presented here may be suitable for the chemical characterization of other distilled liquors.

## Methods

### Materials

*n*-Pentane, 2-octanol (internal standard), and standards of hexanoic acid, ethyl hexanoate, butyric acid, ethyl butyrate, heptanoic acid, ethyl heptanoate, ethyl valerate, ethyl lactate, ethyl acetate, 1-butanol, 1-hexanol, isopentyl alcohol, 2-phenylethanol and furfural were purchased from Aladdin Industrial Corporation (Shanghai, China). Sodium chloride, redistilled diethyl ether, and anhydrous sodium sulphate were purchased from Sino-pharm Chemical Reagent Co., Ltd (Shanghai, China). The solid-phase microextraction (SPME) fiber was purchased from Supelco (Bellefonte, PA, USA). Water was purified from a Milli-Q system.

### Chinese liquors

A total of eighteen types of Luzhoulaojiao liquors were supplied from Luzhou Laojiao Co., Ltd (Sichuan, China). For avoiding the bias and making our sampling more reasonable, Eighteen types of Luzhoulaojiao liquors were randomly chosen and labelled as L1 ~ L18. Xifeng, Fen, and Moutai were purchased in local supermarket. All these Chinese liquors were transported to Shanghai Jiao Tong University (Shanghai, China) for further analysis.

### GC × GC/TOF-MS analysis

The comprehensive two-dimensional gas chromatography/time-of-flight mass spectrometry (GC × GC) system consists of an Agilent 7890 GC equipped with a TOF-MS (Pegasus 4D, Leco Corporation, USA) used to acquire mass spectral data from the GC × GC. The GC oven of this system contains two capillary columns (Table 6). The oven was kept at 60°C for 1 min at first, followed by an increase of 5°C/min to 165°C, then to a final temperature of 280°C at 25°C/min and held for 14 min. The mass spectrometer was operated at an acquisition rate of 100 spectra per second, ranging from 20 to 400 u. The electron impact ionization energy was 70 eV and the acquisition voltage was 1,700 V. The temperature for the ion source was set as 220°C.

DB-5MS (0.25 μm, J&W Scientific) and DB-17HT (0.1 μm, J&W Scientific) GC columns were chosen to enhance the chemical analysis (Table 6). The first separation took place on a conventional nonpolar GC column (DB-5MS). On this column the analytes were separated mainly according to their vapour pressure as they would be in a conventional one-dimensional GC system. Then the first-column eluate was separated into a large number of adjacent small fractions. Each of these was focused and subsequently re-injected into the second, medium GC column (DB-17HT). The connection between two GC columns (a nonpolar column and a medium column) is a typical combination in GC × GC, which could produce ordered and orthometric chromatograms^15^. According to the papers^7^,^38^, for the fast separation of GC × GC resulting in narrow peaks with widths of 120–600 ms, only the TOF-MS was qualified to acquire ≥ 50 spectra/second for reconstruction of the chromatogram and for the quantification.

### Liquid-liquid extraction (LLE)

LLE was done according to the description in literature^5^. In brief, each Luzhoulaojiao liquor sample (40 mL) was concentrated into 0.5 mL through the process of extraction, washing, dehydration and concentration. The samples previously handled with sodium chloride and saturated sodium chloride solutions were extracted by the mixture of redistilled diethyl ether and *n*-pentane for four times. The organic phase layers were combined and washed with saturated sodium chloride and deionized water, respectively. After that, anhydrous sodium sulphate was used to dry the extracts for whole night and then all the extracts were filtered and concentrated for further analysis.

### Solid-phase microextraction (SPME) parameters

A DVB/CAR on PDMS (divinylbenzene/carboxen on polydimethylsiloxane, 50/30 μm) fiber was used to extract the liquor samples. The SPME fiber was conditioned at 250°C for one hour, as directed by the manufacturer. Each liquor sample (5 mL) was diluted with Milli-Q water (15 mL) to an appropriate concentration of ethanol to decrease the limit of comprehensive detection for acetones and aldehydes^16^ and then saturated with sodium chloride. Ten milliliters of each liquor sample was put in the 20 mL vial capped with a PTFE septum and aluminium cap. The solution was incubated for 15 min and then extracted for 45 min at 50°C in a thermostatic bath. After that, the fiber was desorbed in the inlet of the GC prior for chemical analysis.

### Quantification methods

The sample preparation and analysis methods were carried out as above. The peak area of target compounds and internal standard were summed up. The standard curves for individual compounds were built up plotting the response ratio of target compound and internal standard against the concentration ratio. Each standard (0.5 g) listed in the part of materials was weighed accurately, and all the standards were transferred into a 50- mL volumetric flask for making the standard solution (10 g/L) with water-ethanol (water:ethanol = 1:1) as solvent. Then the standard solutions of different concentrations were generated by different gradient dilutions, and proper internal standard (2-octanol) was also added, respectively, when making the dilutions. After LLE and SPME (the extraction methods are the same as the sample preparation), the standard solutions were analyzed by GC × GC/TOF-MS.

## Author Contributions

F.T., F.Y. and P.X. conceived and designed the experiments. F.Y. and Y.L. performed the experiments. F.Y., Y.L. and F.T. analyzed the data. B.Y., C.S., Z.L. and P.X. contributed reagents, materials and analysis tools. F.Y., F.T. and P.X. wrote the paper. All authors have read and approved the final manuscript.

## Supplementary Material

Supplementary Information Supplementary information

Supplementary Information Dataset 1

Supplementary Information Dataset 2

## Figures and Tables

**Table 1:** Part of compounds detected in Luzhoulaojiao liquor L9

Compound	Area %	Similarity	Reverse	Library
Ethyl hexanoate	2.837	909	909	REPLIB
Ethyl lactate	1.815	944	946	REPLIB
Ethyl acetate	1.709	908	913	REPLIB
Hexanoic acid	0.698	953	953	REPLIB
Ethyl butyrate	0.215	936	936	REPLIB
Butanoic acid	0.045	869	869	MAINLIB
Ethyl valerate	0.032	937	937	REPLIB
Heptanoic acid	0.031	900	901	REPLIB
Ethyl heptanoate	0.023	930	933	REPLIB
Furfural	0.024	945	946	MAINLIB
1-Hexanol	0.004	887	919	REPLIB
Phenylethyl Alcohol	0.002	844	909	REPLIB

**Table 2:** Quantification of some abundant and bioactive compounds in Luzhoulaojiao liquor L9

Compound	Concentration (mg/L)
Ethyl hexanoate	2,221 ± 12
Ethyl acetate	693 ± 8
Ethyl lactate	316 ± 4
Hexanoic acid	300 ± 111
Butanoic acid	109.7 ± 0.7
Ethyl butyrate	46.3 ± 0.8
Heptanoic acid	36 ± 1
Furfural	30.96 ± 0.05
Ethyl valerate	10.7 ± 0.1
Phenylethyl Alcohol	3.66 ± 0.03
Ethyl heptanoate	3.38 ± 0.04
1-Hexanol	2.76 ± 0.02
1-Butanol	1.784 ± 0.005

**Table 3:** Part of distinct markers in Luzhoulaojiao liquor L9

Compound	Area %	Similarity	Reverse	Library
Butyl caprylate	0.0016	802	822	MAINLIB
Furfuryl hexanoate	0.0029	763	858	MAINLIB
Octadecane	0.0003	843	855	REPLIB
Undecanoic acid	0.0002	658	722	REPLIB
Phenylethyl butyrate	0.0135	828	893	MAINLIB
Hexadecanoic acid methyl ester	0.0189	874	881	REPLIB
17-Octadecynoic acid	0.0009	673	741	MAINLIB
Undecane	0.0090	896	921	REPLIB
Dodecanoic acid, ethyl ester	0.0011	797	850	REPLIB
Heptanoic acid	0.0334	793	797	REPLIB

**Table 4:** Bioactive compounds common to 18 types of Luzhoulaojiao liquors^20^,^21^,^22^,^23^,^24^,^25^,^26^,^27^,^28^,^29^,^30^,^31^,^32^,^33^,^34^,^35^,^36^,^37^

Compound	Extraction Method
2,4-Decadienal	LLE
Acetophenone	SPME
Nonanal	SPME
Felbamate	LLE
Tetramethylpyrazine	SPME
5-Amino-2-methyl-2H-tetrazole	LLE
1-Butanol, 3-methyl-, acetate	SPME
Phenylethyl alcohol	LLE
Phenylethyl butyrate	SPME, LLE
Heptanoic acid	SPME, LLE
Octanoic acid	LLE
Nonanoic acid	LLE
Undecanoic acid	LLE
Hexadecanoic acid	LLE
Hexanoic acid ethyl ester	SPME, LLE
Heptanoic acid ethyl ester	SPME, LLE
Octanoic acid ethyl ester	SPME, LLE
Nonanoic acid ethyl ester	LLE
Decanoic acid ethyl ester	LLE
Undecanoic acid ethyl ester	LLE
Dodecanoic acid ethyl ester	LLE
Tetradecanoic acid ethyl ester	SPME, LLE
Hexadecanoic acid ethyl ester	LLE
9,12,15-Octadecatrienoic acid ethyl ester	LLE

**Table 5:** Part of aroma compounds common to 18 types of Luzhoulaojiao liquors

Aroma compound	
1-Butanol	1-Butanol, 3-methyl-
1-Pentanol	2-Heptanol
1-Hexanol	Acetic acid
1-Heptanol	Propanoic acid
1-Octanol	Propanoic acid, 2-methyl-
Butanoic acid	Butanoic acid, 2-methyl-
Butanoic acid, 3-methyl-	Pentanoic acid
Hexanoic acid	Phenylethyl alcohol
Heptanoic acid	Phenol, 4-ethyl-
Octanoic acid	Nonanoic acid
Ethyl acetate	Ethyl pentanoate
Ethyl butanoate	

**Table 6:** Column sets conditions

Column Set	First column	Second column
Stationary phase	DB-5MS	DB-17HT
Length (m)	29.950	1.640
Int. Diameter (mm)	0.25	0.1
Max Temp (°C)	340	360
Film thickness (μm)	0.25	0.1
Bleed Masses	73 149 207 281	73 149 207 281
