# Supplementary material for: Chemical Analysis of the Chinese Liquor Luzhoulaojiao by Comprehensive Two-Dimensional Gas Chromatography/Time-of-Flight Mass Spectrometry
Source: Sci Rep. 2015 Apr 10;5:9553. doi: 10.1038/srep09553 (PMC4392506; doi:10.1038/srep09553)
Supplement: Supplementary Information [file srep09553-s1.doc]

Supplementary information

Chemical Analysis of the Chinese Liquor Luzhoulaojiao by Comprehensive Two-Dimensional Gas Chromatography/Time-of-Flight Mass Spectrometry

Feng Yao^1^, Bin Yi^2^, Caihong Shen^2^, Fei Tao^1§^, Yumin Liu^3^, Zhixin Lin^1^, Ping Xu^1^

^1^State Key Laboratory of Microbial Metabolism, and School of Life Sciences & Biotechnology, Shanghai Jiao Tong University, Shanghai, People’s Republic of China

^2^National Engineering Research Center of Solid-State Brewing, Luzhou, People’s Republic of China

^3^The Instrumental Analysis Center, Shanghai Jiao Tong University, Shanghai 200240, People’s Republic of China

^§^Address correspondence to: Fei Tao, School of Life Sciences and Biotechnology, Shanghai Jiao Tong University, Shanghai 200240, PR China. Tel: +86-21-34206647; Fax: +86-21-34206723; E-mail: taofei@sjtu.edu.cn

**Supplementary Information: Different classes of common compounds in 18 types of Luzhoulaojiao liquor**

**Supplementary Table S1. Common alcohols in 18 types of Luzhoulaojiao** liquor

| **Alcohol** | |
| --- | --- |
| 3-Methyl-2-butanol | 2-Chloroethanol |
| 2-Pentanol | 2-Heptanol |
| 6, 9-Pentadecadien-1-ol | 2-Methyl-5-hexen-3-ol |
| 1,6-Heptadien-4-ol | 3-Buten-2-ol, 2-methyl- |
| 1,6-Octadien-3-ol, 3,7-dimethyl- | 3-Cyclohexen-1-ol, 5-methylene-6-(1-methylethenyl)-  3-Hexanol, 2,5-dimethyl- |
| 1-Butanol |
| 1-Butanol, 3-methyl- |
| 1-Heptanol | 3-Pentanol, 2,4-dimethyl- |
| 1-Hexanol | 6-Pentadecanol |
| 1-Nonanol | Hexane-1,3,4-triol, 3,5-dimethyl- |
| 1-Nonen-4-ol | p-Cresol |
| 1-Octanol | Phenol, 4-[2-(methylamino)ethyl]- |
| 1-Pentanol | Phenol, 4-ethyl- |
| 1-Propanol, 2-methyl- | Phenylethyl Alcohol |

**Supplementary Table S2. Common Organic acids in 18 types of Luzhoulaojiao** liquor

| **Organic Acid** | |
| --- | --- |
| 2-Thiopheneacetic acid | Hydrocinnamic acid |
| 3,3-Dimethylacrylic acid | Nonanoic acid |
| Acetic acid | Octanoic acid |
| Acetic acid, hydroxy- | Pentanoic acid |
| Benzeneacetic acid | Phenyllactic acid |
| Butanoic acid | Propanedioic acid, propyl- |
| Butanoic acid, 2-methyl- | Propanoic acid |
| Butanoic acid, 3-methyl- | Propanoic acid, 2-methyl- |
| Heptanoic acid | Undecanoic acid |
| Hexadecanoic acid |  |

**Supplementary Table S3. Common esters in 18 types of Luzhoulaojiao** liquor

| **Ester** |  |
| --- | --- |
| 9-Octadecenoic acid ethyl ester | Furfuryl hexanoate |
| 1-Butanol, 3-methyl-, acetate | Heptanoic acid, 3-methylbutyl ester |
| 2(3H)-Benzofuranone, 3-methyl- | Heptanoic acid, ethyl ester |
| 2(3H)-Furanone, 5-butyldihydro- | Heptyl hexanoate |
| 2(3H)-Furanone, dihydro-5-pentyl-  2(4H)-Benzofuranone, 5,6,7,7a-tetrahydro-4,4,7a-trimethyl- | Hex-5-enoic acid, ethyl ester |
|  | Hexadecanoic acid, ethyl ester |
|  | Hexadecanoic acid, methyl ester |
| 2-Butenoic acid, ethyl ester  2-Furancarboxylic acid, ethyl ester  2-Hexenoic acid, ethyl ester | Hexanedioic acid, bis(2-ethylhexyl) ester |
|  | Hexanoic acid, 2-methylpropyl ester |
|  | Hexanoic acid, 3-hydroxy-, ethyl ester |
| 2-Methylpentyl hexanoate | Hexanoic acid, butyl ester |
| 3-Hexenoic acid, ethyl ester | Hexanoic acid, ethyl ester |
| 4-Heptenoic acid, ethyl ester | Hexanoic acid, hexyl ester |
| 5-Heptenoic acid, ethyl ester | Hexanoic acid, methyl ester |
| 9,12,15-Octadecatrienoic acid, ethyl ester | Hexanoic acid, pentyl ester |
| 9,12-Octadecadienoic acid, methyl ester | Isobutyl acetate |
| Acetic acid, 2-phenylethyl ester | Isopentyl hexanoate |
| Acetic acid, diethoxy-, ethyl ester | Methoxyacetic acid, pentyl ester |
| Acetic acid, dimethoxy-, methyl ester | Nonanoic acid, 9-oxo-, ethyl ester |
| Benzeneacetic acid, ethyl ester | Nonanoic acid, ethyl ester |
| Benzenepropanoic acid, ethyl ester | Octanoic acid, 3-methylbutyl ester |
| Benzoic acid, 2-methylpropyl ester | Octanoic acid, ethyl ester |
| Benzoic acid, ethyl ester | Oxalic acid, allyl nonyl ester |
| Butanedioic acid, diethyl ester | Pentanedioic acid, diethyl ester |
| Butanedioic acid, ethyl 3-methylbutyl ester | Pentanoic acid, 3-methylbutyl ester |
| Butanoic acid, 2-furanylmethyl ester | Pentanoic acid, 2-hydroxy-4-methyl-, ethyl ester |
| Butanoic acid, 2-hydroxy-3-methyl-, ethyl ester |  |
| Butanoic acid, 2-methyl-, ethyl ester | Pentanoic acid, 4-methyl-, ethyl ester |
| Butanoic acid, 2-methylpropyl ester | Pentanoic acid, ethyl ester |
| Butanoic acid, 3-methyl-, ethyl ester | Phenylacetic acid, cyclobutyl ester |
| Butanoic acid, ethyl ester | Phenylethyl butyrate |
| Butanoic acid, propyl ester | Propanoic acid, 1-methylpropyl ester |
| Butyl caprylate | Propanoic acid, 2-hydroxy-, ethyl ester |
| Butyl citrate | Propanoic acid, 2-methyl-, 2-phenylethyl ester |
| Cyclohexanecarboxylic acid, ethyl ester |  |
| Decanoic acid, ethyl ester | Propanoic acid, 2-methyl-, 3-methylbutyl ester |
| Dodecanoic acid, ethyl ester |  |
| Ethyl 9-hexadecenoate | Propanoic acid, 2-methyl-, ethyl ester |
| Ethyl Acetate | Propanoic acid, ethyl ester |
| Ethyl 2-pentenoate | Tetradecanoic acid, ethyl ester |
| Formic acid, ethenyl ester |  |

**Supplementary Table S4. Common ketones, aldehydes and acetals in 18 types of Luzhoulaojiao** liquor

| **Ketone** | |
| --- | --- |
| 2-Buten-1-one, 1-(2,6,6-trimethyl-1,3-cyclohexadien-1-yl)- | 2-Octanone |
|  | 2-Octen-4-one |
| 1-Propanone, 1-(2-furanyl)- | 2-Pentanone |
| 2-Acetyl-5-methylfuran | 2-Undecanone, 6,10-dimethyl- |
| 2-Butanone | 3-Penten-2-one |
| 2-Dodecanone | Acetophenone |
| 2-Heptanone | Cyclopentanone |
| 2-Hexanone, 3-methyl-4-methylene- | Ethanone, 1-(2-furanyl)- |
| 2-Nonanone | Ethanone, 2,2-dihydroxy-1-phenyl- |
| 2-Buten-1-one, 1-(2,6,6-trimethyl-1,3-cyclohexadien-1-yl)- | Tricyclo[4.2.2.0(1,5)]decan-4-one |
|  |  |
| **Aldehyde** | |
| 2,4-Decadienal | Benzeneacetaldehyde, ethylidene- |
| 2-Furancarboxaldehyde, 5-methyl- | Butanal, 2-methyl- |
| 2-Octenal | Butanal, 3-methyl- |
| Acetaldehyde, hydroxy- | Furfural |
| Acetaldehyde, methoxy- | Hexanal |
| Benzaldehyde | Nonanal |
| Benzeneacetaldehyde |  |
| **Acetal** | |
| 1-Propene, 3,3-diethoxy- | Heptane, 1,1-diethoxy- |
| 2-Furaldehyde diethyl acetal | Hexane, 1,1-diethoxy- |
| Benzene, (2,2-diethoxyethyl)- | Nonane, 1,1-diethoxy- |
| Butane, 1,1-diethoxy-3-methyl- | Octane, 1,1-diethoxy- |
| Ethane, 1,1-diethoxy- | Propane, 1,1-diethoxy- |

**Supplementary Table S5. Common nitrogen/sulfur-containing compounds in 18 types of Luzhoulaojiao** liquor

| **Nitrogen-containing compound** | |
| --- | --- |
| 1-[(2-Thienylcarbonyl)oxy]-2,5-pyrrolidinedione | Glycine |
| 1-Azaspiro[5,5]undecane | Heptanonitrile |
| 1-Butanamine, N-butylidene- | N,N-Dimethyldodecanamide |
| 1-Naphthalenamine, N-phenyl- | Naphthalene-2-ol, 1-(2-methylphenyliminomethyl)- |
| 3-Amino-5-t-butylisoxazole |
| 5-Amino-2-methyl-2H-tetrazole | Octanenitrile |
| 9-Octadecenamide | Pentanenitrile |
| Benzoic acid, hydrazide | Propanedinitrile, bicyclo[3.3.1]non-9-ylidene- |
| Benzonitrile | Pyrazine, 2,6-dimethyl- |
| Butane, 1-nitro- | Pyrazine, tetramethyl- |
| Dodecanamide | Pyrazine, trimethyl- |
| Ethanol, 2-nitro- | Pyridine |
| Ethanone, 2-(5-nitrotetrazol-2-yl)-1-phenyl- | Pyridine-3-carboxamide, 1,2-dihydro-4,6-dimethyl-2-thioxo- |
| Felbamate |
| Formamide, N-methoxy- | Pyridine, 3-(1-methyl-2-pyrrolidinyl)- |
| **Sulfur-containing compound** | |
| Butanethioic acid, S-methyl ester | Sulfurous acid, 2-ethylhexyl isohexyl ester |
| Disulfide, dimethyl | Sulfurous acid, cyclohexylmethyl dodecyl ester |
| Disulfide, pentyl propyl |
| Furan, 2-[(ethylthio)methyl]- | Trisulfide, dipropyl |
